# The potential role of Hippo pathway regulates cellular metabolism via signaling crosstalk in disease-induced macrophage polarization

**DOI:** 10.3389/fimmu.2023.1344697

**Published:** 2024-01-11

**Authors:** Yina An, Shuyu Tan, Jingjing Yang, Ting Gao, Yanjun Dong

**Affiliations:** ^1^ National Key Laboratory of Veterinary Public Health Security, College of Veterinary Medicine, China Agricultural University, Beijing, China; ^2^ College of Veterinary Medicine, China Agricultural University, Beijing, China

**Keywords:** macrophage polarization, inflammatory diseases, metabolism, Hippo, regulatory network

## Abstract

Macrophages polarized into distinct phenotypes play vital roles in inflammatory diseases by clearing pathogens, promoting tissue repair, and maintaining homeostasis. Metabolism serves as a fundamental driver in regulating macrophage polarization, and understanding the interplay between macrophage metabolism and polarization is crucial for unraveling the mechanisms underlying inflammatory diseases. The intricate network of cellular signaling pathway plays a pivotal role in modulating macrophage metabolism, and growing evidence indicates that the Hippo pathway emerges as a central player in network of cellular metabolism signaling. This review aims to explore the impact of macrophage metabolism on polarization and summarize the cell signaling pathways that regulate macrophage metabolism in diseases. Specifically, we highlight the pivotal role of the Hippo pathway as a key regulator of cellular metabolism and reveal its potential relationship with metabolism in macrophage polarization.

## 1 Introduction

**Figure 1:**
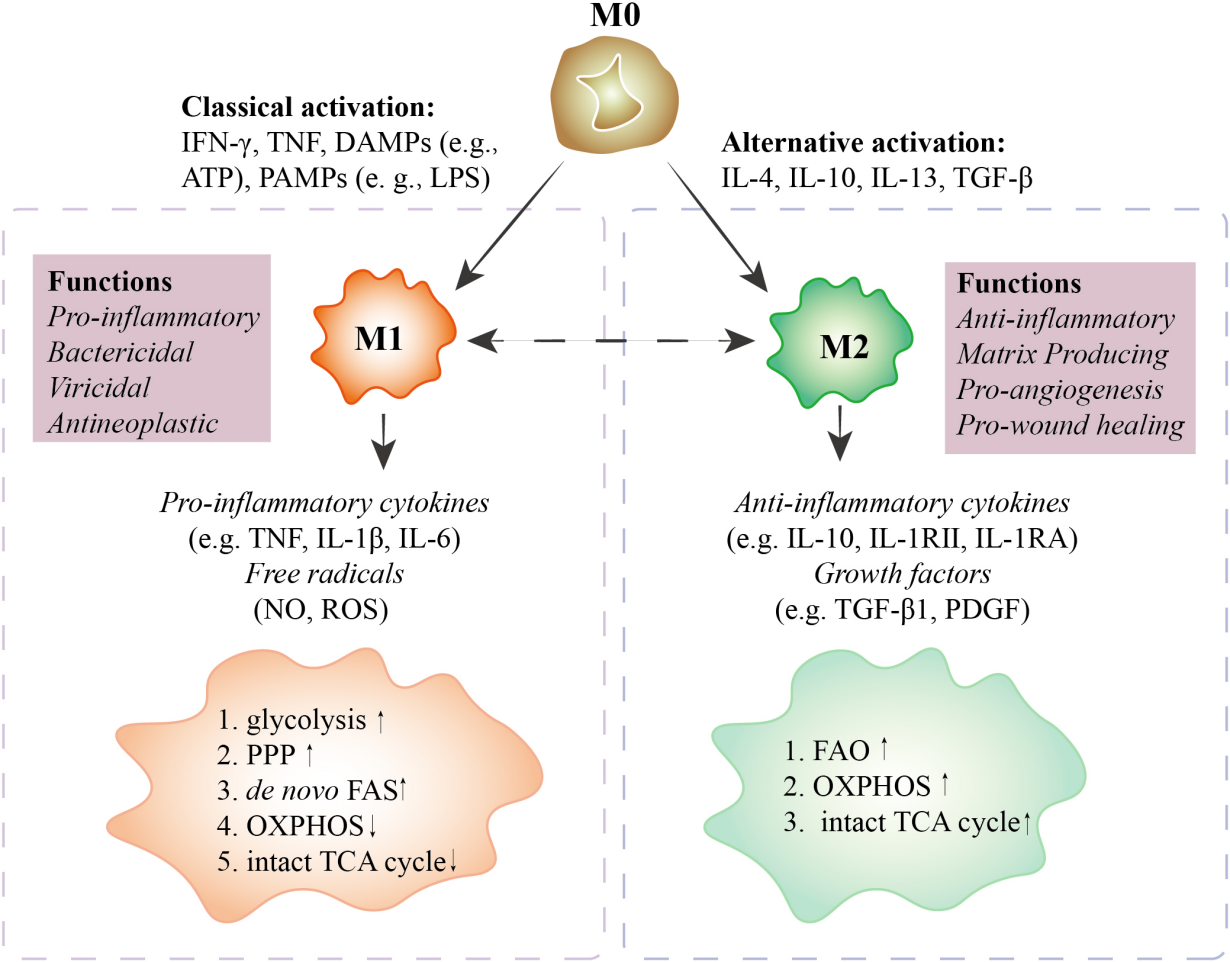
Characteristics of polarized macrophages. M0 macrophages polarize into M1 macrophages with IFN-γ, TNF, DAMPs (e.g., ATP), and PAMPs (e.g., LPS) stimuli. M1 macrophages secrete pro-inflammatory cytokines (e.g., TNF, IL-1β, IL-6) and free radicals (NO, ROS) to perform pro-inflammatory, bactericidal, viricidal, and antineoplastic activities. In M1 macrophages, glycolysis, PPP, and *de novo* FAS are upregulated, and OXPHOS and the intact TCA cycle are downregulated. M0 macrophages polarize into M2 macrophages with IL-4, IL-10, IL-13, and TGF-β treatment. M2 macrophages secrete anti-inflammatory cytokines (e.g., IL-10, IL-1RII, IL-1RA) and growth factors (e.g., TGF-β1, PDGF) to perform anti-inflammatory, matrix-producing, pro-angiogenesis, and pro-wound healing functions. In M2 macrophages, FAO, OXPHOS, and the TCA cycle are upregulated. In special cases, M1 can be polarized toward M2. However, whether M2 can be polarized to M1 is still debated. M0 macrophages, unactivated macrophages; M1 macrophages, classically activated macrophages; M2 macrophages, alternatively activated macrophages; IFN, interferon; TNF, tumor necrosis factor; DAMPs, damage-associated molecular patterns; PAMPs, pathogen-associated molecular patterns; PPP, pentose phosphate pathway; OXPHOS, oxidative phosphorylation; FAS, fatty acid synthesis; FAO, fatty acid oxidation; TCA, tricarboxylic acid cycle; IL, interleukin; NO, nitric oxide; ROS, reactive oxygen species; TGF, transforming growth factor; PDGF, platelet-derived growth factor.

**Figure 2:**
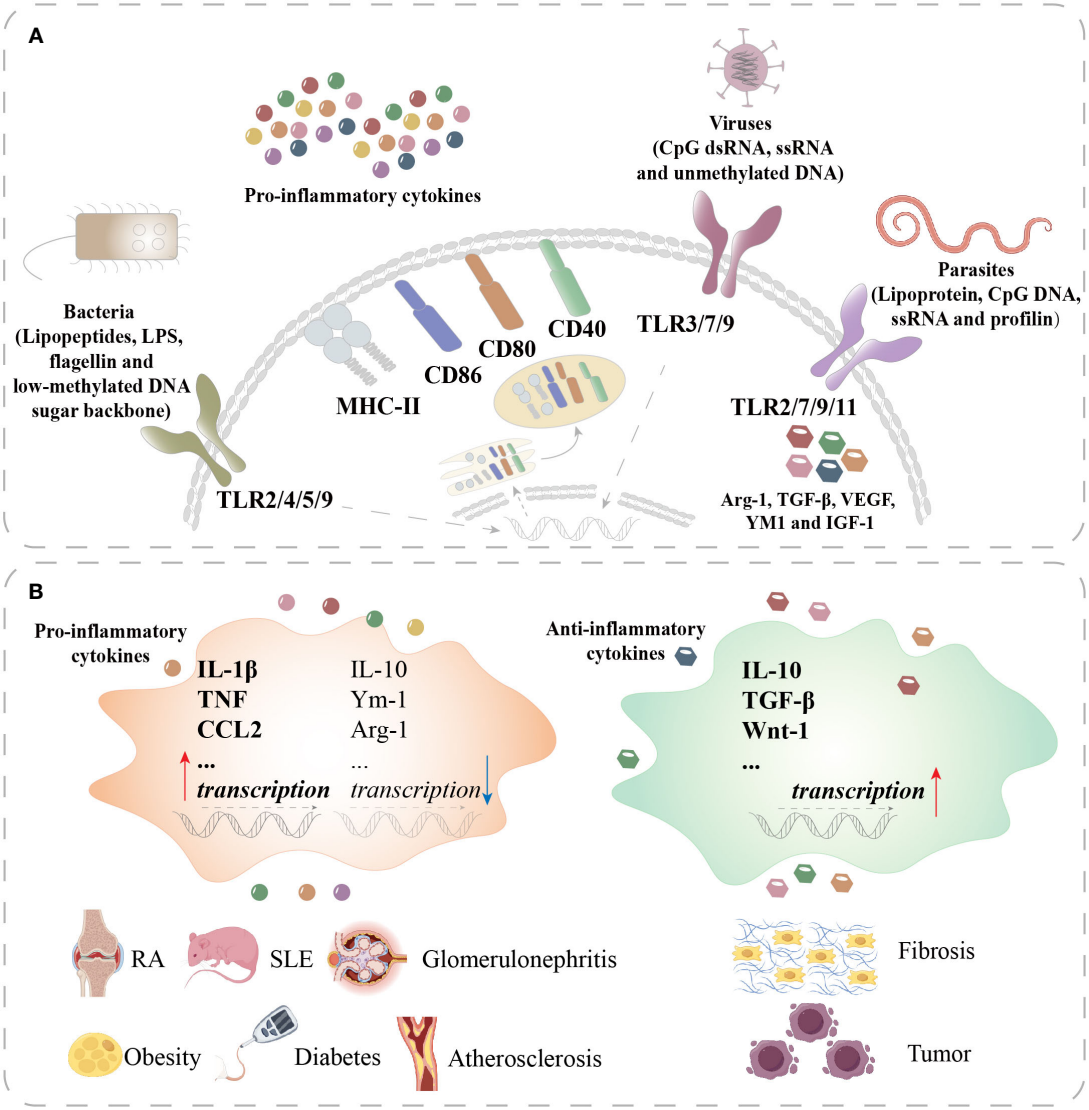
Macrophage polarization in different diseases. **(A)** Macrophages recognize lipopeptides, LPS, flagellin, and the low-methylated DNA sugar backbone of bacteria through TLR2/4/5/9 and recognize CpG dsRNA, ssRNA, and unmethylated DNA of viruses through TLR3/7/9 to activate M1 macrophages. Activated M1 macrophages secrete proinflammatory cytokines and upregulate MHC-II, CD86, CD80, and CD40. Macrophages recognize lipoprotein, CpG DNA, and ssRNA and profilin of parasites through TLR2/7/9/11 to polarize into M2 macrophages. M2 macrophages produce Arg-1, TGF-β, VEGF, YM1, and IGF-1. **(B)** In RA, SLE, glomerulonephritis, obesity, diabetes, and atherosclerosis, macrophages are overactivated to upregulate the transcription of proinflammatory cytokines such as IL-1β, TNF, and CCL2 and downregulate the transcription of anti-inflammatory cytokines such as IL-10, Ym-1, and Arg-1. In fibrosis and tumors, M2 macrophages are overactivated to upregulate anti-inflammatory cytokines such as IL-10, TGF-β, and Wnt-1. TLR, toll-like receptor; MHC, major histocompatibility complex; CD, cluster of differentiation; VEGF, vascular endothelial growth factor; IGF, insulin-like growth factor; CCL, chemokine cc-motif ligand; RA, rheumatoid arthritis; SLE, systemic lupus erythematosus.

**Figure 3:**
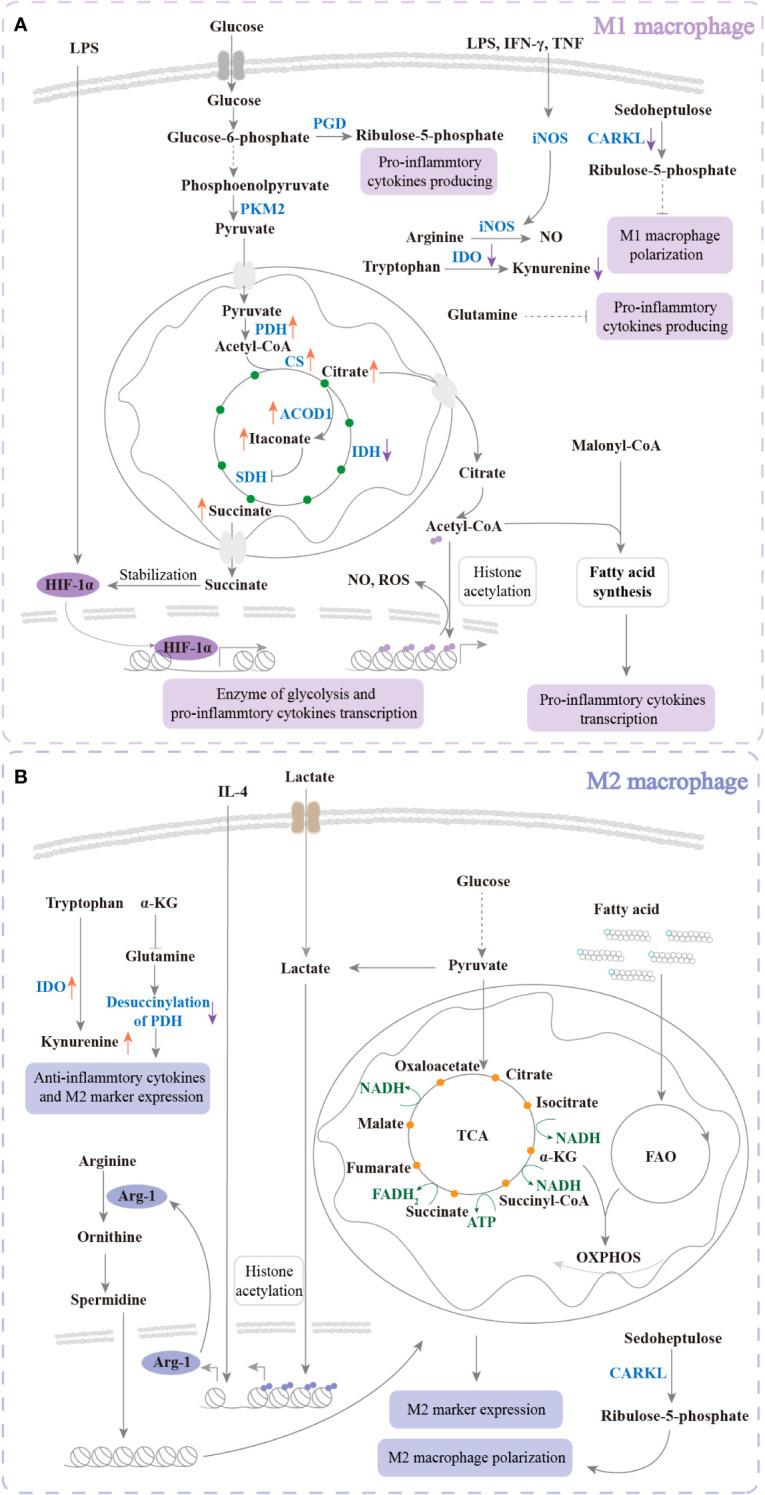
The metabolism of regulated polarization. **(A)** Under LPS, IFN-γ, or TNF treatment, glycolysis, PPP, and FAS are upregulated to increase M1 polarization. The TCA cycle and amino acid metabolism are changed. The changes promote intermediate products of TCA and NO production of large quantities to enhance pro-inflammation and kill bacteria. Glucose uptake is increased and further produces pyruvate. Pyruvate enters mitochondria and promotes citrate, itaconate, and succinate accumulation. Citrate enters the cytoplasm to produce acetyl-CoA, which participates in histone acetylation and FAS. Succinate enters the cytoplasm to maintain the stabilization of HIF-1α. HIF-1α translocates into the nucleus to transcribe glycolysis enzymes and proinflammatory cytokines. In addition, IDO is downregulated to inhibit kynurenine production to increase M1 polarization. Ribulose-5-phosphate and CARKL are decreased to inhibit M1 polarization. Glutamine plays a negative role in pro-inflammatory cytokine production. **(B)** Response to IL-4, TCA cycle, FAO, and OXPHOS is upregulated for M2 polarization. Amino acid metabolism is changed to enhance anti-inflammatory cytokine production. The intermediate products of TCA and FAO are involved in OXPHOS. In response to IL-4 and lactate, M2 markers such as Arg-1 are transcribed. Arg-1 promotes arginine catabolism and further enhances TCA and OXPHOS. In addition, IDO is upregulated to increase kynurenine production. Ribulose-5-phosphate and CARKL are increased. These changes enhance M2 polarization. Glutamine plays a positive role in M2 polarization. PK, pyruvate kinase; PGD, phosphogluconate dehydrogenase; PDH, pyruvate dehydrogenase complex hyperacetylation; CS, citrate synthase; ACOD, aconitase decarboxylase; SDH, succinate dehydrogenase; IDH, isocitrate dehydrogenase; iNOS, inducible nitric oxide synthase; IDO, indoleamine 2,3-dioxygenase; HIF, hypoxia-inducible factor; NAD, nicotinamide adenine dinucleotide; ATP, adenosine 5’-triphosphate.

**Figure 4:**
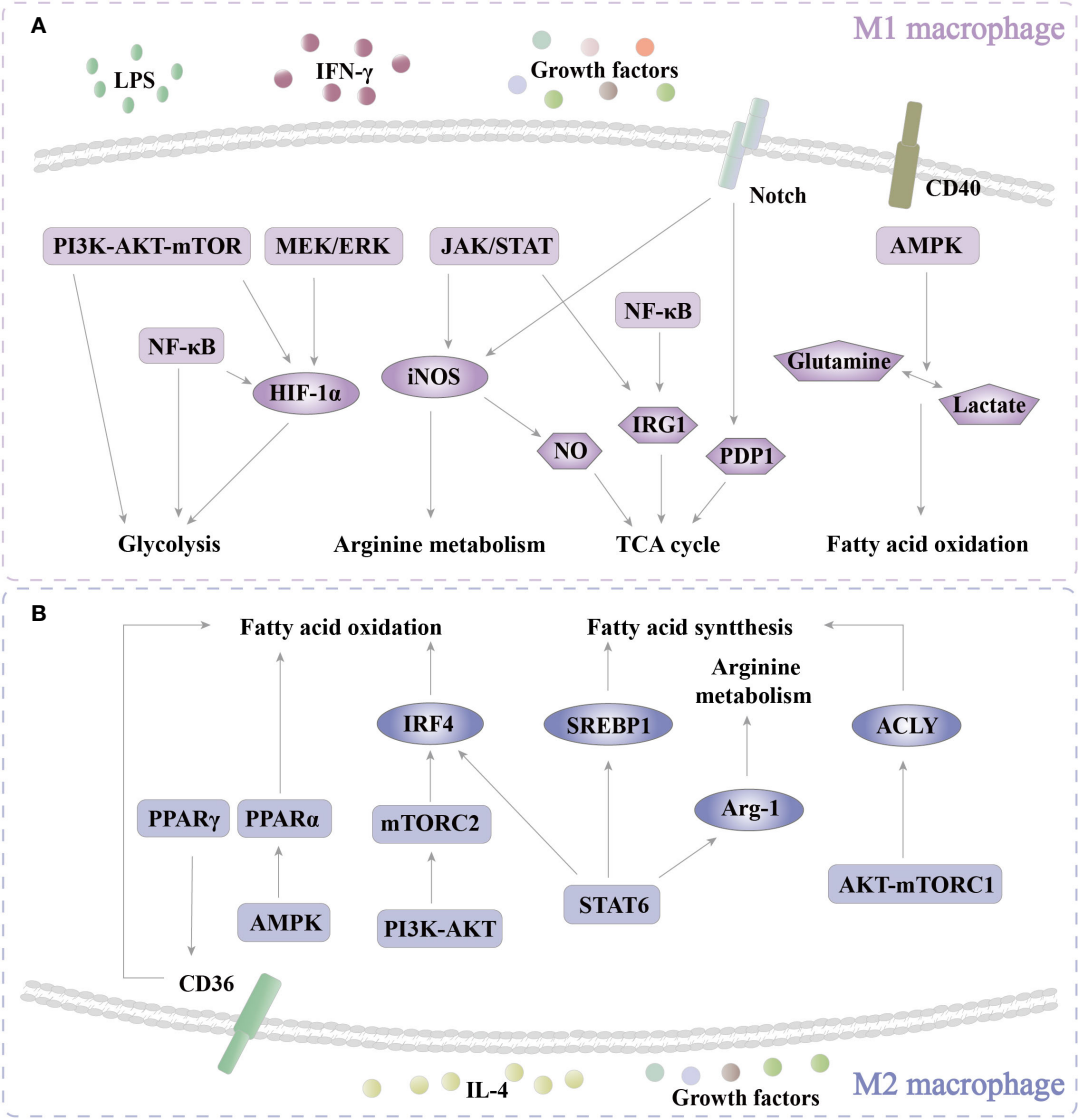
Pathways of regulatoy metabolism. **(A)** In M1 polarization, PI3K-AKT-mTOR, MEK/ERK, and NF-κB are activated to enhance glycolysis through HIF-1α. In addition, PI3K-AKT-mTOR and NF-κB also directly regulate glycolysis. JAK/STAT increases arginine metabolism by iNOS. NO that is produced by the enzymolysis of iNOS participates in the TCA cycle. JAK/STAT and NF-κB regulate the TCA cycle through IRG1. Notch affects the TCA cycle via PDP1. CD40-AMPK enhances FAO by regulating the conversion of glutamine and lactate. **(B)** In M2 polarization, PPARγ enhances FAO by increasing CD36 expression. AMPK/PPARα promotes FAO. PI3K-AKT-mTORC2 increases the level of FAO via IRF4. STAT6 boosts FAO, FAS, and arginine metabolism via IRF4, SREBP1, and Arg-1, respectively. AMPK-mTORC1 increases FAS by ACLY. PI3K, phosphoinositide 3-kinase; mTOR, mammalian target of rapamycin; NF-κB, nuclear factor kappa-B; MEK, mitogen-activated extracellular signal-regulated kinase; ERK, extracellular regulated protein kinase; JAK, Janus kinase; STAT, signal transducer and activator of transcription; AMPK, adenosine 5’-monophosphate (AMP)-activated protein kinase; PPAR, peroxisome proliferator-activated receptor.

**Figure 5:**
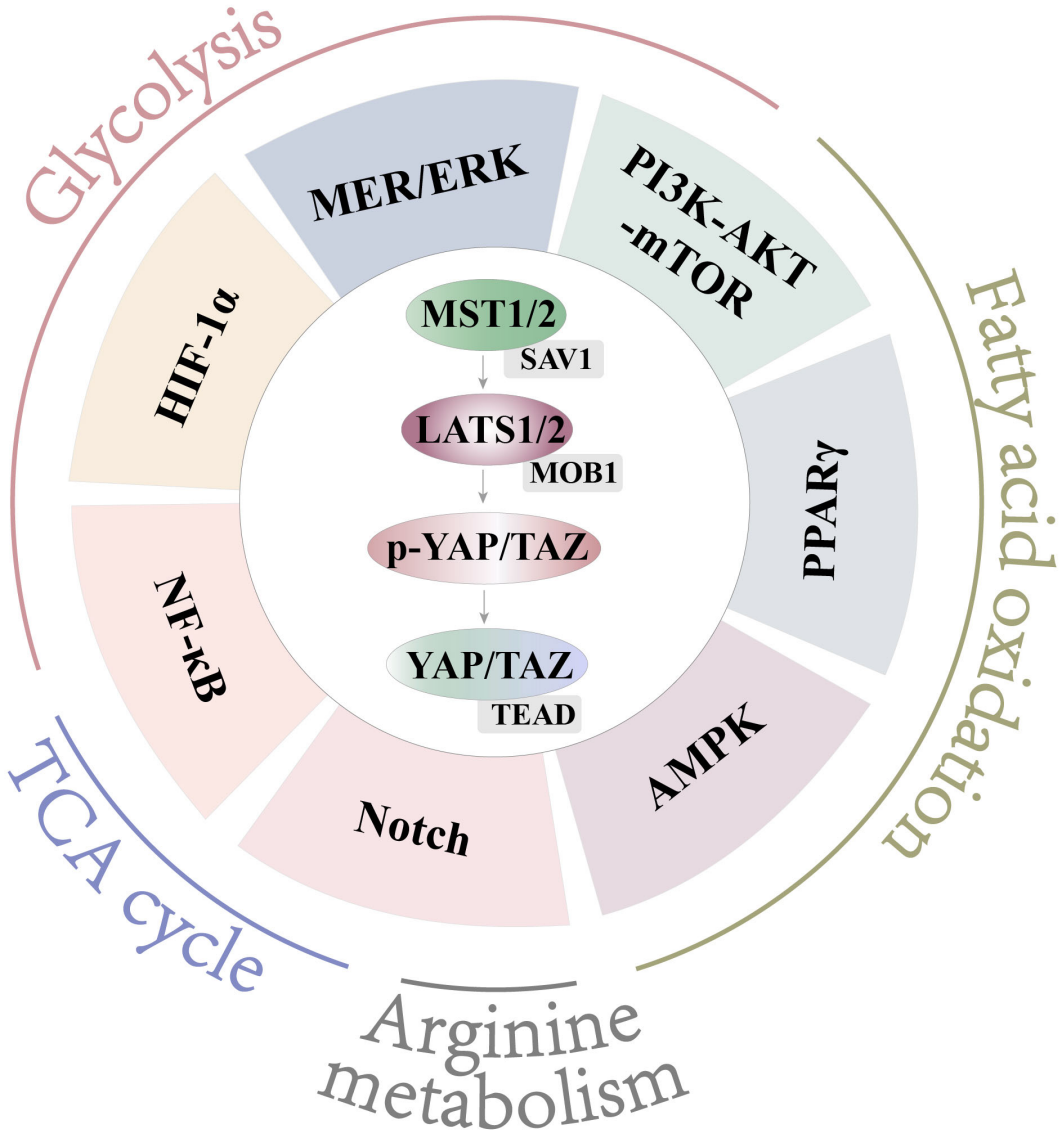
Crosstalk with Hippo and other pathways. Hippo engages in crosstalk with NF-κB, HIF-1α, MEK/ERK, and PI3K-AKT-mTOR to affect glycolysis. Hippo engages in crosstalk with PI3K-AKT-mTOR, PPARγ, and AMPK to affect FAO. Hippo engages in crosstalk with Notch to affect arginine metabolism. Hippo engages in crosstalk with Notch and NF-κB to affect the TCA cycle.

Macrophages are critical components of innate immunity and play a vital role in homeostasis, tissue repair, and defense against bacterial, viral, and neoplastic threats. These cells are highly plastic and can be polarized into distinct phenotypes. The M1 phenotype produces proinflammatory cytokines and free radicals for antibacterial, antiviral, and antineoplastic responses. In contrast, the M2 phenotype secretes anti-inflammatory cytokines that are involved in antiparasitic, proangiogenic, and prowound healing responses (1, 2) (**Figures 1**, **2**). Recent studies have highlighted the importance of cellular metabolism in regulating macrophage polarization and controlling inflammatory responses. Metabolism, such as glycolysis, pentose phosphate pathway (PPP), tricarboxylic acid cycle (TCA cycle), and fatty acid synthesis (FAS), influence the M1 polarization (**Figure 3A**), which is associated with bacterial infection, obesity, diabetes, fatty liver, and atherosclerosis (**Figure 2**). Conversely, the intact TCA cycle, fatty acid oxidation (FAO), and FAS affect the M2 polarization (**Figure 3B**), which is related to parasite infection, tumors, and fibrosis (2, 3) (**Figure 2**). Furthermore, various signaling pathways, including mammalian target of rapamycin (mTOR)-phosphoinositide 3-kinase (PI3K)-AKT, peroxisome proliferator-activated receptor (PPAR) γ, adenosine 5’-monophosphate (AMP)-activated protein kinase (AMPK), mitogen-activated extracellular signal-regulated kinase (MEK)/extracellular regulated protein kinase (ERK), and Notch, have been implicated in regulating macrophage metabolism (**Figure 4**). However, these studies are scattered and lack a comprehensive overview. By summarizing the literature, we found that the Hippo pathway is a central player in the cellular metabolic signaling network. It has the potential to intertwine with these pathways to form a metabolic regulatory network that affects macrophage metabolism (4–6) (**Figure 5**).

Understanding the mechanisms underlying metabolic regulation is critical for preventing and treating inflammatory diseases. The Hippo pathway could represent a novel target for modulating the metabolic polarization of macrophages and holds promise for therapeutic interventions in inflammatory diseases. This comprehensive review aims to explore the role of the Hippo pathway-mediated cellular metabolism and reveals the potential regulatory relationship of Hippo signaling with cellular metabolism in macrophage polarization.

## 2 Macrophage polarization-related diseases

Macrophages are important immune cells that exhibit remarkable plasticity and can switch two main phenotypes: M1 and M2. M1 macrophages are activated by damage-associated molecular patterns (DAMPs) or pathogen-associated molecular patterns (PAMPs) and combat bacterial, viral, and neoplastic threats (**Figures 1**, **2**). M2 macrophages are activated by cytokines such as IL-4, IL-10, IL-13, and transforming growth factor-β (TGF-β) and promote anti-inflammatory responses, tissue repair, and angiogenesis (**Figures 1**, **2**). The balance and switch between M1 and M2 macrophages are crucial for maintaining homeostasis and controlling disease processes.

### 2.1 Infectious diseases

Normally, microbial infection triggers macrophage polarization and the inflammatory response by recognizing PAMPs. Common infectious diseases are caused by bacterial, viral, and parasitic infections.

During bacterial infection, pattern recognition receptors (PRRs) are anchored on the surface of macrophages to recognize PAMPs of bacteria, such as toll-like receptor (TLR) 2, TLR4, TLR5, and TLR9, which are specific for lipopeptides, lipopolysaccharide (LPS), flagellin, and the low-methylated DNA sugar backbone, respectively (7, 8) (**Figure 2A**). PAMPs induce macrophages to express high-level costimulatory molecules, such as cluster of differentiation (CD) 40, CD80, CD86, and major histocompatibility complex-II (MHC-II), to perform antigen presentation (2) (**Figure 2A**). These activated macrophages also produce and secrete proinflammatory cytokines to promote the Th1 immune response (2) (**Figure 2A**). Some bacteria could decrease the level of M1 polarization for bacterial survival. *Mycobacterium tuberculosis* (*M. tuberculosis*) inhibits pro-inflammation and induces M2 polarization (9). In addition, we found that *Mobile colistin resistance (mcr)*-1/3 resistance enzyme-modified LPS decreases NF-κB activation and induces weaker macrophage response than native LPS *in vitro*. *mcr-1/3* positive bacteria induce lower level macrophage inflammation and M1 polarization, further causing higher mortality in mice (10). Wu reported that promoting M2 polarization and decreasing M1 polarization ameliorate bacterium-induced acute lung injury (11).

Viral infection triggers a pro-inflammatory response that is similar to bacterial infection. TLR 3, 7, and 9 identify CpG motifs-dsRNA, ssRNA, and unmethylated DNA of viruses, respectively (12) (**Figure 2A**). Jiang found that simian immunodeficiency virus (SIV) induces M2 macrophage polarization (13). Hepatitis B virus (HBV) decreases the levels of IL-1β and IL-6 secretion by M1 macrophages, and increases the IL-10 secretion by M2 macrophages (14).

Parasite infection causes M2 polarization. TLR2, 7, 9, and 11 identify lipoprotein, CpG DNA, ssRNA, and profilin of parasites (15) (**Figure 2A**). Helminth infection induces a Th2 immune response to produce high levels of Arg-1, TGF-β, vascular endothelial growth factor (VEGF), YM1, and insulin-like growth factor-1 (IGF-1) to inhibit parasitic confinement and clearance (16) (**Figure 2A**). Protozoans induce anti-inflammatory responses to antagonize macrophages for protozoan survival (17).

Therefore, enhancing the proinflammatory response in the microenvironment can enhance bacterial and viral clearance, although this may cause more severe tissue damage. M2 anti-inflammation increases parasitic clearance. Those evidences suggest that regulating the balance of M1 and M2 activation in infectious disease could be a potential therapeutic intervention. It is of great significance to explore the molecular mechanism of the polarization balance between M1 and M2 macrophages to control microbial infection.

### 2.2 Noninfectious diseases

Abnormal macrophage polarization also affects noninfectious diseases. Aberrant M1 activation induces autoimmune disease and chronic inflammatory diseases. M2 overactivation is involved in fibrosis and tumors.

#### 2.2.1 M1 activation-related disease

Autoimmune diseases feature a macrophage-mediated uncontrolled and overinflammatory immune response, including rheumatoid arthritis (RA), systemic lupus erythematosus (SLE), and glomerulonephritis (**Figure 2B**). In these diseases, a large number of proinflammatory factors are mainly secreted by M1 macrophages (**Figure 2B**). Obesity, diabetes, and atherosclerosis are chronic inflammatory diseases (4). In obesity, adipocytes secrete proinflammatory cytokines and free fatty acids (FFAs) that induce the recruitment of monocytes to polarize M1 macrophages. M1 macrophage-mediated proinflammatory cytokine production amplifies the proinflammatory circuit and leads to insulin resistance (18). In atherosclerosis, macrophages uptake oxidized lipids to develop into foam cells and further release proinflammatory cytokines, which induce low-level inflammation in the arterial wall (19).

Therefore, reducing the level of M1 polarization and enhancing M2 polarization are essential strategies for treating autoimmune diseases and chronic inflammatory diseases.

#### 2.2.2 M2 activation-related disease

M2 macrophages can suppress the proinflammatory response, release anti-inflammatory cytokines, promote tissue repair, and secrete extracellular matrix (ECM) for wound healing. However, persistent stimuli can lead to dysregulation of this process, resulting in excessive ECM deposition, upregulation of myofibroblast activity, and a chronic inflammatory environment with infiltration of M2 macrophages and other immune cells (2) (**Figure 2B**). M2 macrophages not only play critical roles in tissue repair and fibrosis but also promote the occurrence and development of tumors. Tumor-associated macrophages (TAMs) generally display the M2 phenotype and secrete anti-inflammatory cytokines and growth factors to promote the proliferation, invasion, and metastasis of tumor cells and inhibit the antitumor immune response (20, 21) (**Figure 2B**).

Therefore, the regulation of macrophage polarization level is of great significance for controlling ECM accumulation and tumor cell proliferation and metastasis.

## 3 Cellular metabolism-mediated macrophage polarization

Previously, we have summarized the important role of macrophage polarization in disease. We will further discuss the factors that regulate macrophage polarization. Many studies have shown that metabolic pathways can regulate macrophage polarization. Reprogramming the functions of macrophages by changing metabolism has become a consensus for regulating macrophage polarization diseases. Next, we will summarize how metabolism affects polarization.

### 3.1 Glycolysis

Glycolysis metabolizes glucose to produce pyruvate, lactate, and ATP (4). Although glycolysis only produces a small amount of ATP, it proceeds rapidly to provide energy for requiring rapid production of physiological activities (4). In response to LPS or PAMPs, the metabolism of macrophages switches toward anaerobic glycolysis, but IL-4 almost does not affect glycolysis (22). Hypoxia-inducible factor-1α (HIF-1α) is a transcription factor. In response to LPS stimulation, HIF-1α is upregulated and promotes glycolytic enzyme and proinflammatory cytokine expression (23, 24) (**Figure 3A**). Inhibition of macrophage glycolysis can reduce M1 polarization by facilitating the proteasome degradation of HIF-1α (25, 26). Pyruvate kinase (PK) is the rate-limiting enzyme of glycolysis. Enhancing glycolysis by increasing PKM2 phosphorylation or decreasing PKM2 ubiquitination can promote polarization of M1 macrophages, following an increased proinflammatory response to aggravate liver fibrosis (27) (**Figure 3A**). Annexin A5 promotes hepatic macrophage shifting from M1 to M2 by inhibiting PKM2 phosphorylation (28). Lactate (a product of anaerobic glycolysis and glutaminolysis)-derived acetylation in histone lysine residues directly induces transcription of M2-like genes such as Arg-1 at the late phase of M1 polarization with bacterial infection (29) (**Figure 3B**). Lactate inhibits the levels of M1 markers and the proportion of M1 macrophages in breast cancer tissues (30).

These results show that enhancing glycolysis can promote M1 polarization. Notably, HIF-1α and the rate-limiting enzymes are crucial in increasing M1 macrophage polarization (**Figure 3A**). Meanwhile, lactate inhibits the M1 marker and promotes M2 polarization (**Figure 3B**). This suggests that glycolysis is a target to regulate macrophage polarization.

### 3.2 PPP

As a branch of glycolysis, the PPP is a major source of NADPH and is required for ribonucleotide synthesis (31). The PPP is significantly upregulated in M1 macrophages, suggesting that the PPP plays an important role in proinflammatory polarization (3, 32, 33). Glucose-6-phosphate (G6P, an intermediate product of glycolysis) is oxidized to produce NADPH, which is the foundation of macrophage-killing activity and is involved in fatty acid biosynthesis and antioxidant defense mechanisms (34). Inhibiting PPP by the knockdown of 6-phosphogluconate dehydrogenase (PGD) can decrease macrophage-producing pro-inflammatory cytokines with LPS treatment (35) (**Figure 3A**). In addition, the carbohydrate kinase-like protein CARKL, which catalyzes an orphan reaction in PPP, can reprogram M1- and M2- polarization metabolism (36) (**Figures 3A, B**). CARKL reduces the production of proinflammatory cytokines (M1 phenotype) (36). *CARKL* gene overexpression in macrophages significantly inhibits intracellular ROS production and is more sensitive to M2 stimulation (37).

Those evidences suggest that the PPP is also involved in increasing proinflammatory cytokine production and M1 macrophage polarization.

### 3.3 The TCA cycle

Pyruvate in the cytoplasm is transported to the mitochondria and then used in the TCA cycle (also known as the Krebs cycle) (4). (**Figure 3A**). In M1 macrophages, isocitrate dehydrogenase (IDH, which catalyzes the conversion of isocitrate to α-ketoglutarate) breaks the TCA cycle (38), resulting in the accumulation of the intermediate products citrate, itaconate, and succinate, which participate in pro-inflammation (34, 38–44). HBV decreases the incomplete TCA cycle and drives M2 polarization (45). After DCA (an agonist of TCA) treatment, enhancing incomplete TCA can significantly reduce M2 marker expression (45). Inhibition of citrate synthase (CS)/pyruvate dehydrogenase complex hyperacetylation (PDH) in macrophages increases M2-like polarization (45). (**Figure 3A**). Citrate is exported from mitochondria and catabolized to produce acetyl-CoA to enhance glycolytic gene transcription by histone acetylation, which drives M1 activation (39) and inflammatory cytokine, nitric oxide (NO) and reactive oxygen species (ROS) production (40) (**Figure 3A**). Inhibition of the citrate carrier (CIC, which exports citrate from mitochondria) ensures oxidative metabolic flux in the TCA cycle (39). CIC inhibition decreases *IL-1β* and *Nos 2* gene (M1 marker) expression, increases *IL-10* and *Arg-1* gene (M2 marker) expression, and promotes the switch from M1 to M2 (39). The accumulation of itaconate has been recognized as characteristic of inflammation and the killing activity of M1 macrophages (41). In M1 macrophages, itaconate is produced in mitochondria through aconitase decarboxylase 1 (ACOD1) (42) (**Figure 3A**). It has been shown that itaconate enhances innate antibacterial immunity (43). In addition, itaconate decreases IL-1β, IL-6, IL-12, and IL-18 levels by inhibiting succinate dehydrogenase-mediated oxidation of succinate after LPS treatment (44). Succinate has been reported to be involved in M1 activation after LPS stimuli (40). Succinate can stabilize HIF-1α and drive IL-1β secretion by promoting glycolysis (23) (**Figure 3A**).

Therefore, intact TCA can completely metabolize pyruvate, enhance oxidative phosphorylation, and promote macrophages to polarize into M2. Interrupted TCA can increase the pro-inflammatory response. These results suggest that the polarization and biological activity of macrophages can be regulated by affecting the integrity of the TCA cycle.

### 3.4 Fatty acid metabolism

Nonlipid precursors (e.g., glucose-derived citrate) are transported to the cytosol and synthesized as Ac-CoA (the raw material for fatty acid synthesis) (46). When fatty acids are broken down, they produce Ac-CoA, FADH2, and NADH in the mitochondrial matrix (46). Fatty acid metabolism also provides energy for supporting the macrophage inflammatory response and polarization (47). FAS plays a vital role in energy source and prostaglandin biosynthesis in M1 macrophages (48), and malonyl-CoA promotes the pro-inflammatory response (49, 50) (**Figure 3A**). FAO (also known as β oxidation) in M2 macrophages can drive mitochondrial oxidative phosphorylation (OXPHOS) to produce energy. Inhibition of FAO prevents M2 polarization (51) (**Figure 3B**). However, other studies have shown that FAO is not necessary for M2 polarization upon IL-4 stimulation, and FAO may play a role in M1 activity (52–54), suggesting that the regulation of macrophage polarization by fatty acid metabolism may be more complicated than previously thought. Other functions of FAO are receiving increasing attention. Chandra reported that when FAO is inhibited in macrophages, the bacteria cannot survive, and the tissue bacterial load of mice decreases (55). Enhancing FAO exacerbates LPS-induced sepsis (56).

Consequently, fatty acid metabolism affects macrophage polarization. However, there are many contradictions in the literature. How fatty acid metabolism regulates polarization still needs to be further explored.

### 3.5 Amino acid metabolism

Amino acid metabolism usually refers to the synthesis of amino acids and their decomposition (4). Amino acid and their metabolic producers are important for the immune response (34). A deficiency of amino acid may affect the migration, division, and maturation of immune cells (34). Deficiency of glutamine increases M1-specific marker genes and decreases M2-specific markers (38) (**Figures 3A, B**). α-Ketoglutarate (α-KG) produced by glutamate decomposition promotes M2 polarization but inhibits M1 polarization (57) (**Figure 3B**). The limiting enzyme of tryptophan metabolism is indoleamine 2,3-dioxygenase (IDO) (34). Macrophages polarize into M2 with overexpression of IDO, and silencing IDO macrophages polarize into M1 (58) (**Figures 3A, B**). Two L-arginine catalytic enzymes, inducible nitric oxide synthase (iNOS) and Arg-1, are M1 and M2 macrophage markers, respectively. Arginine metabolism plays an essential role in macrophage inflammatory function (59). In response to proinflammatory stimuli (e.g., LPS, IFN-γ, and TNF), iNOS catabolizes arginine to produce NO. NO promotes M1 polarization and prevents M1 to M2 polarization (60, 61) (**Figure 3A**). In M2 macrophages, Arg-1 catabolizes arginine to produce ornithine and urea, and ornithine is catalyzed to produce spermidine. Furthermore, TCA and OXPHOS are promoted, which induces macrophage polarization to M2 (62) (**Figure 3B**). However, the two catabolic pathways of arginine are not independent of each other. In tumors, arginine is catabolized by both iNOS and Arg-1, which suggests that targeting arginine is a new strategy to regulate macrophage polarization (63).

These results indicate that amino acids, related enzymes, and their production can change the polarization of macrophages. Therefore, they can be used as targets to explore the mechanism of macrophage polarization.

## 4 The regulation of macrophage metabolism

According to the above summary, macrophage metabolism is an important way to regulate macrophage polarization. These metabolic pathways are affected by different cellular signals. Here, we will summarize the specific signaling pathways involved in regulating macrophage metabolism, aiming to identify the potential mechanisms regulating macrophage metabolism.

### 4.1 The regulatory mechanisms of M1 metabolism

As we have shown before, glycolysis, amino acid metabolism, the TCA cycle, and FAO regulate M1 polarization. Therefore, we focused on these metabolic pathways to explore the signaling pathways affecting M1 metabolism.

In glycolysis, there is a critical transcription factor, HIF-1α, which transcribes the key glycolysis kinases hexokinase 1/2 (HK1/2), glucose transporters 1/3 (GLUT1/3), lactate dehydrogenase (LDHA) and PKM (64). Therefore, controlling the expression and activation of HIF-1α is a strategy for affecting glycolysis. In breast cancer cells, responses to ROS, PI3K and AKT are activated and further promote HIF-1α transcription of HK2, thereby enhancing macrophage glycolysis and enhancing tumor survival (65). A similar process also exists in macrophages. In macrophages of mice with *E. coli*-induced sepsis, triggering receptors expressed on myeloid cells-1 (TREM-1), PI3K, AKT, and mTOR are activated in a cascade (66). Then, HIF-1α translocates into the nucleus and initiates the transcription of GLUT1, HK2, and LDHA to enhance glycolysis, control the activation of NOD-like receptor thermal protein domain associated protein 3, and affect the secretion of inflammatory factors in macrophages (66) (**Figure 4A**). In addition, the PI3K-AKT-mTOR pathway is also activated to regulate HIF-1α by growth factors (64). MEK/ERK, downstream of growth factors and LPS, can also restrict glycolysis through HIF-1α (67, 68) (**Figure 4A**). With LPS induction, another classical inflammatory pathway, nuclear factor kappa-B (NF-κB), is also involved in regulating the upregulation of glycolysis (69). In macrophages, inhibition of NF-κB activation damages glycolysis activity (**Figure 4A**). As a transcriptional regulator, NF-κB can participate in the transcriptional regulation of HK2 in CD8^+^ T cells, which is also a way for NF-κB to inhibit glycolysis (70). This suggests that PI3K-AKT-mTOR, MEK/ERK, and NF-κB promote M1 polarization by enhancing glycolysis.

In macrophages, signal transducer and activator of transcription (STAT) regulates L-arginine metabolism. Under IFN-γ treatment, Janus kinase (JAK)/STAT1 is phosphorylated to transcript *Nos2* mRNA and express iNOS for L-arginine metabolism (71, 72) (**Figure 4A**). Furthermore, in the gastric cancer microenvironment, the SLC2A3-STAT3-SLC2A3 feedback loop promotes macrophage phenotype transition by phosphorylating downstream glycolytic targeting genes (73). This suggests that the STAT family plays an important role in the macrophage metabolism regulatory mechanism. When bacteria infect macrophages, immune-responsive gene 1 (IRG1), which is needed for the decarboxylation of cis-aconitate to form itaconate in the TCA cycle, is upregulated, therefore inducing itaconate accumulation to damage microbes (74, 75) (**Figure 4A**). However, prolonged excessive accumulation of itaconate leads to decreased sensitivity of M1 macrophages (75). In addition, IRG1 encodes an emerging IFN regulatory protein (IRP) with antimicrobial activity (74), and both JAK/STAT and NF-κB are upstream of IRG1 (**Figure 4A**). In a zebrafish bacterial infection model, the glucocorticoid receptors and JAK/STAT signaling pathways cooperatively regulate IRG1 expression (76). Therefore, IRG may be used as new targets to explore new antibacterial mechanisms and develop new antibacterial drugs in macrophages. Those evidences indicate JAK/STAT, which is related to the TCA cycle, increases M1 polarization. NF-κB affects the TCA cycle to promote M1 polarization.

In response to LPS stimuli, the Notch pathway is activated to initiate transcription of the *Nos2* and *pyruvate dehydrogenase phosphatase 1* (*Pdp1*) genes to induce concurrent glucose flux to the TCA cycle for M1 activation (77) (**Figure 4A**). In M1, a large amount of NO damages the mitochondrial respiratory chain (78). In cells lacking NO, citrate is significantly decreased, and itaconate and succinate are increased (78). These results indicate that NO is involved in regulating metabolic processes such as the TCA cycle in M1 cells and may participate in proinflammatory and antibacterial processes through metabolism (**Figure 4A**).

Different from LPS, CD40 signaling can regulate the conversion of glutamine and lactate to affect the NAD/NADH ratio by AMPK, thereby reprogramming the FAO-induced proinflammatory response and antitumorigenic activation (79) (**Figure 4A**).

Therefore, PI3K-AKT-mTOR, MEK/ERK, and NF-κB promote M1 polarization by enhancing glycolysis. JAK/STAT regulates arginine metabolism by iNOS expression to increase M1 polarization. NF-κB plays a proinflammatory role in the TCA cycle. Notch increases M1 polarization by the TCA cycle. CD40-AMPK reprograms the FAO-induced proinflammatory response. In summary, PI3K-AKT-mTOR, MEK/ERK, NF-κB, JAK/STAT, Notch, CD40-AMPK, and NO-related pathways are involved in the metabolic regulation of macrophage M1 polarization (**Figure 4A**).

### 4.2 The regulatory mechanisms of M2 metabolism

After reviewing the literature, we found that FAS, FAO, and arginine metabolism play central roles in regulating M2 polarization. Therefore, we focused on exploring the signaling pathways that affect M2 metabolism.

PPARγ, a hallmark of M2 macrophages, is involved in lipid metabolism by upregulating CD36 (fatty acid transporter) to promote fatty acid uptake and FAO (80) (**Figure 4B**). PPARα is also involved in regulating macrophage metabolism. Li reported that in DSS-induced ulcerative colitis (UC), activation of AMPK-PPARα can promote β-oxidation, decrease *de novo* lipogenesis, and promote M2 polarization of macrophages (81) (**Figure 4B**). However, this study did not clearly show that AMPK-PPARα changes occurred in macrophages. However, based on previous reports that AMPK, PPARα, and FAO are upregulated under the induction of IL-4+IL-13 (51, 81), we speculated that a similar process would occur in macrophages. With IL-4 or MCSF induction, IRF4 is transcribed to promote FAO and OXPHOS by inducing glucose metabolism. The study also reported that STAT6 and mTORC2 are the pathways regulated upstream of IRF4 (82) (**Figure 4B**). In summary, PPARγ, AMPK-PPARα, and PI3K-AKT-mTORC2 increase M2 polarization by enhancing FAO.

STAT6 also regulates FAS. In response to IL-4, the anabolic transcription factor sterol regulatory element binding protein 1 (SREBP1) is activated by STAT6 to trigger *de novo* FAS and then promotes macrophage M2 activation (83) (**Figure 4B**). A study established that AKT-mTORC1 is also involved in FAS by activating ATP citratelyase (ACLY, catalyze citrate to produce acetyl-CoA) to promote acetyl-CoA derived acetylation of histone and M2 gene transcription (84) (**Figure 4B**). Under IL-4 stimuli, STAT6 is activated to produce Arg-1 for L-arginine metabolism (71) (**Figure 4B**). In summary, STAT6 and AKT-mTORC1 promote M2 polarization by FAS, and STAT6 also affects arginine metabolism for M2 polarization.

PPARγ, AMPK-PPARα, and PI3K-AKT-mTORC2 increase M2 polarization by regulating FAO. STAT6 promotes M2 polarization through FAO and arginine metabolism. AKT-mTORC1 increases M2 polarization by FAS. This suggests that these pathways are targets to control M2 macrophage polarization by affecting metabolism (**Figure 4B**).

It is worth noting that these metabolic and cell signaling pathways always crosstalk each other. Therefore, we speculated that there might be a pathway that could bring these complex regulatory mechanisms together.

## 5 The potential relationship of Hippo pathway with the regulatory pathway of metabolism

The cellular signaling pathways that influence macrophage metabolism are relatively dispersed. There is a lack of center pathways to crosstalk those pathways into a network. Through reviewing the literature, we found that the Hippo pathway engages in crosstalk with multiple cellular signaling pathways. The signaling network will help us better explore the potential relationship of Hippo signaling with the regulatory pathway of metabolism in macrophages.

The Hippo pathway is widespread and cross-interacts with a large number of pathways to perform different functions. The Hippo pathway was first identified in *Drosophila*, and it controls organ size by regulating cell proliferation and apoptosis. The Hippo pathway consists of a series of kinases, mainly including mammalian Sterile 20-like kinases 1/2 (MST1/2), SAV1, large tumor suppressor (LATS1/2), MOB1, Yes-associated protein (YAP), transcriptional coactivator with PDZ-binding motif (TAZ), and TEAD1-4. In response to a variety of stimuli, the Hippo pathway is activated or inhibited. When the Hippo pathway is activated, MST1/2 (its critical kinase of Hippo) undergoes heterodimerization with SAV (**Figure 5**). MST1/2 is activated and phosphorylated, followed by stepwise phosphorylation of SAV1, LATS1/2, and MOB1 (**Figure 5**). Phosphorylated YAP/TAZ (YAP and TAZ are homologous analogs), the downstream effectors of Hippo, bind to 14-3-3 proteins and are retained in the cytoplasm or are polyubiquitinated and degraded in the cytoplasm. When the Hippo pathway is off, YAP/TAZ in the cytoplasm translocate into the nucleus and bind to transcription factors or TEADs to initiate the transcription of downstream factors (**Figure 5**). YAP/TAZ regulates cell proliferation, differentiation, apoptosis, and other biological processes (85). In the Hippo pathway, MST1/2 is a key regulatory kinase, and YAP is a downstream effector molecule. Thus, most studies have focused on MST1/2 and YAP.

The Hippo pathway plays an important role in cell proliferation and differentiation. YAP aggravates inflammatory bowel disease (IBD) by balancing M1/M2 polarization (86). With LPS or IL-4+IL-13 stimuli, transcription regulatory YAP is activated (86). Activated YAP increases IL-6 expression to promote M1 polarization and decreases p53 expression to disrupt M2 polarization (86). In myocardial infarction, it has also been reported that YAP promotes pro-inflammation by secreting IL-6 and impairs the reparative response by decreasing Arg-1 expression (87). During acute liver injury and acute lung injury, inhibition of YAP promotes M2 macrophage polarization to decrease proinflammatory cytokine production and impair tissue injury (88–90). This evidence shows that YAP promotes M1 polarization and decreases M2 polarization to affect inflammatory diseases. In bacterial infection mice, deficiency of MST1/2 in macrophages increases inflammatory damage in the lung and promotes proinflammatory cytokine secretion (91). We highlight above that Hippo is an important pathway in regulating macrophage polarization.

These evidences show the Hippo pathway is an important controller in macrophage polarization (86, 91). Metabolism is also an important factor in regulating macrophage polarization (4, 92). But, at present, no study directly evidences that Hippo can affect macrophage polarization by regulating metabolism. This part of the research is still a gap. Therefore, exploring whether the Hippo pathway regulates macrophage polarization by affecting metabolism is a new and meaningful research direction. According to the reports, Hippo can regulate cellular metabolism in tumors, hepatocytes, cardiomyocytes, and chondrocytes (93–97). And the AKT-mTOR, PPARγ, MEK/ERK, AMPK, NF-κB, HIF-1α, and Notch pathways play important roles in macrophage metabolism (64–69, 77, 80, 81). Meanwhile, we also found that the Hippo pathway is in crosstalk with these regulatory pathways in macrophages or other cells (74, 90, 96, 98–105). Therefore, we comprehensively reviewed the literature to summarize the crosstalk between Hippo and metabolic regulatory pathways in different kinds of cells. Then, we further speculated on the potential relationship of the Hippo pathway with the regulatory pathway of metabolism in macrophages. As a transcription regulator, YAP itself can mediate the transcription of key metabolic kinases. Therefore, we speculate the Hippo pathway may regulate cellular metabolism either directly or through crosstalk with these pathways.

### 5.1 The potential relationship of Hippo pathway with the regulatory pathway of glycolysis

The Hippo pathway and glycolysis regulate M1 polarization (22, 86–90). However, no literature directly suggests that Hippo regulates macrophage polarization by glycolysis. These studies have reported that MST1 and YAP affect glycolysis in tumors, hepatocytes, cardiomyocytes, and chondrocytes (93–96). Activation of MST1 inhibits glycolysis by reducing the expression of GLUT1 and C-MYC in tumors, while YAP has a positive correlation with C-MYC, GLUT3, HK2, and PFKB3 to increase glycolysis in hepatocytes (93, 94). In cardiomyocytes, YAP promotes glycolysis by upregulating GLUT1 (95). In chondrocytes, YAP also plays a positive role in glycolysis (96). Therefore, YAP and MST1/2 may play roles in glycolysis to regulate macrophage polarization. But that needs to be further proven using experimental proof.

That reports that HIF-1α, MEK/ERK, PI3K-AKT-mTOR, and NF-κB regulate M1 polarization by glycolysis (64–70). Many studies show the Hippo pathway is in crosstalk with these pathways (96, 98–100). Therefore, Hippo, which is in crosstalk with these pathways, may be involved in the regulation of macrophage glycolysis. Next, we will describe specifically how Hippo interacts with these pathways in other cells and macrophages.

First, we summarized the crosstalk between Hippo and these pathways in cells other than macrophages. An important molecule is HIF-1α in glycolysis. It has already been mentioned in many studies that MST1 and YAP are involved in the regulation of HIF-1α in granulocyte progenitor cells, chondrocytes, and hepatocellular carcinoma cells (96, 98, 99). In granulocyte progenitor cells, deficient of MST1 increases glycolysis by enhancing mTOR and HIF-1α (98). During chondrogenesis, YAP binds to HIF-1α to form a complex that promotes glycolysis and chondrogenic differentiation (96). In hepatocellular carcinoma cells, in response to hypoxia, nuclear YAP binds to HIF-1α to maintain the stability of HIF-1α and promote the transcription of glycolytic genes (99). In addition, MEK/ERK also regulates glycolysis (68, 69). YAP inhibits the MEK/ERK pathway in initial segment epithelial cells. MST1/2 at least partially regulates initial segment differentiation by repressing YAP (106). In Jurkat T cells, MEK/ERK can activate MST1, caspase-3/7, and caspase-8 to enhance MST1 proteolytic cleavage (107). In tumor cells, the AKT pathway directly inhibits MST2 activity by promoting MST2/Raf-1 interaction (108). In HEK293 cells, AKT can block MST1 activation by phosphorylating MST1 at S387 (109). Cinar reported that MST1 is cleaved by caspase, and mature MST1 and cleavage products are inhibited by AKT in human prostate cancer cells (110). In idiopathic pulmonary fibrosis, YAP and PI3K-AKT-mTOR interact with each other to regulate the proliferation, migration, and polarity of abnormal cells (111). Therefore, AKT inhibits the activation of MST1/2, and YAP interacts with PI3K-AKT-mTOR. In addition, deficiency of MST1 in granulocyte progenitor cells upregulates glycolysis and downregulates OXPHOS by mTOR and HIF-1α for cell proliferation and differentiation (98). Therefore, according to these evidences, we speculate that Hippo may play a role through crosstalk with HIF-1α and MEK/ERK, and PI3K-AKT-mTOR in promoting glycolysis and the inflammatory response of macrophages.

Next, we summarized the crosstalk between Hippo and these pathways in macrophages. Only a small amount of literature has shown that YAP interacts with NF-κB (100). In M1-type macrophages, YAP enhances the proinflammatory response by binding to NF-κB p65 and increasing the accumulation of p65 in the nucleus (100). Meanwhile, NF-κB plays a role in glycolysis (69). Therefore, we suggest that YAP crosstalk with NF-κB may play a role in glycolysis in macrophages. And it still needs further verification.

Therefore, it can be speculated that YAP affects glycolysis and M1 polarization by p65, HIF-1α, and PI3K-AKT-mTOR (**Figure 5**). MST1 may regulate polarization by glycolysis, which is regulated by AKT (**Figure 5**). However, MEK/ERK function is inconsistent with expectations in glycolysis and M1 polarization as an unknown mechanism. It is worth noting that the above is only our conjecture. However, the role of Hippo in the regulation of glycolysis in macrophages deserves further study.

### 5.2 The potential relationship of Hippo pathway with the regulatory pathway of fatty acid metabolism

The Hippo pathway and fatty acid metabolism regulate M2 polarization (49–54, 86–90). Deletion of YAP/TAZ in brown adipose tissue decreases the oxygen consumption rate and increases body fat content, suggesting that YAP/TAZ plays a role in lipid metabolism (97). They report that PPARγ and AMPK regulate M2 polarization by fatty acid metabolism (80, 81). But no study directly suggests that Hippo is crosstalk with PPARγ and AMPK in macrophages. Most of these literatures focused on epithelial stem cells, adipocytes, and tumor cells (101–104).

To be specific, activation of PPARγ inhibits WB-F344 (rat liver epithelial stem cell) proliferation. It induces cell cycle arrest by regulating the Hippo pathway (including promoting phosphorylation of MST1 and LATS2 and inhibiting nuclear translocation of YAP) (101). In turn, in adipocytes, YAP1 deficiency significantly increases the expression of PPARγ to affect the proliferation and differentiation of ovine preadipocytes (102). AMPK also modulates the activity of the Hippo pathway for energy homeostasis. During glucose starvation, AMPK is activated to phosphorylate YAP in tumor cells (103). Mo also found the same effect in response to energy stress (104). On the one hand, AMPK can phosphorylate LATS1/2 to inhibit YAP. On the other hand, AMPK can directly phosphorylate YAP Ser 94 to inhibit the binding of YAP to TEADs (104). Both PPARγ (a marker for M2) and AMPK-PPARα promote FAO. Therefore, we can speculate that, in the FAO-mediated M2 polarization, Hippo plays a role through crosstalk with PPARγ and AMPK (**Figure 5**).

YAP is inhibited by the PI3K-AKT/β-catenin pathway with IL-4+IL-13 treatment in macrophages (86), and AKT is related to fatty acid metabolism in M2 macrophages (112). Unfortunately, we did not find more evidence about the regulation of fatty acid metabolism by Hippo crosstalk with AKT. The regulation of fatty acid metabolism by Hippo in macrophage polarization is only speculated by us based on the study of other cells, and whether there is such an association still needs to be further verified.

### 5.3 The potential relationship of Hippo pathway with the regulatory pathway of TCA and arginine metabolism in macrophages

The Hippo pathway, TCA, and arginine metabolism regulate macrophage polarization (45, 59–63, 86–91). The Notch pathway promotes the accumulation of TCA intermediates and iNOS (74, 75), and an incomplete TCA cycle and iNOS promote macrophage polarization into M1 macrophages (77). In Kupffer cells, the Notch pathway increases *Yap* gene expression, and YAP can upregulate Notch ligand gene expression involved in macrophage polarization (90). Based on a previous summary, we found that YAP can also promote the TCA cycle through NF-κB in macrophages (74, 100) (**Figure 5**). In addition, in hepatocellular carcinoma, there also is a positive feedback loop between YAP and the Notch pathway (105). These studies suggest that YAP may play a role in M1 polarization by the Notch pathway-mediated accumulation of TCA intermediates and iNOS (**Figure 5**).

In summary, we can make the following assumptions: YAP promotes M1 polarization by glycolysis, incomplete TCA cycle, and arginine metabolism. The involved pathways may be NF-κB, HIF-1α, PI3K-AKT-mTOR, and Notch (**Figure 5**). YAP inhibits M2 polarization by FAO, and the involved pathways may be the PPARγ-, AMPK-, and PI3K-AKT-related pathways (**Figure 5**). In addition, MST1/2 plays a role in macrophage polarization by glycolysis and FAO, which may be regulated by AKT and PPARγ, respectively (**Figure 5**).

Based on the above evidence, there is obvious crosstalk between the Hippo pathway and other pathways that regulate macrophage metabolism, which suggests that the Hippo pathway is a potential target for exploring the mechanism of macrophage metabolism and polarization.

## 6 Discuss: the Hippo pathway and metabolism in macrophage polarization-related diseases

Metabolism can endow macrophages with different functions (92). It is a new treatment idea for diseases to target macrophage polarization by regulating macrophage metabolism (1). Therefore, it is necessary to explore the intrinsic metabolic mechanism in the process of macrophage polarization, especially in the state of diseases. Most studies on metabolic regulation have mainly focused on the PI3K-AKT-mTOR, PPARγ, AMPK, Notch, MEK/ERK, and NF-κB pathways (64–69, 77, 80, 81). However, according to the literature, we found that the Hippo pathway can crosstalk with these metabolic regulatory pathways (74, 90, 96, 98–105). Meanwhile, they reported that regulating the Hippo pathway drives changes in metabolism (93–97). Therefore, we have summarized how the Hippo pathway is in crosstalk with metabolic regulatory pathways. This will facilitate further exploration of the regulatory mechanism of metabolism by the Hippo pathway, which will help us to control the process of metabolic diseases in macrophages. The Hippo pathway is a new potential target that affects macrophage polarization by regulating metabolic pathways and metabolism in macrophages. It is worthy of in-depth exploration.

## Author contributions

YA: Conceptualization, Data curation, Formal analysis, Writing – original draft, Writing – review & editing. ST: Conceptualization, Data curation, Formal analysis, Writing – review & editing. JY: Conceptualization, Data curation, Formal analysis, Writing – review & editing. TG: Conceptualization, Data curation, Writing – original draft, Writing – review & editing. YD: Conceptualization, Funding acquisition, Supervision, Writing – review & editing.
